# Supplementary material for: An open source toolkit for repurposing Illumina sequencing systems as versatile fluidics and imaging platforms
Source: Sci Rep. 2022 Mar 24;12:5081. doi: 10.1038/s41598-022-08740-w (PMC8948189; doi:10.1038/s41598-022-08740-w)
Supplement: Supplementary file 1 — Supplementary Information. [file 41598_2022_8740_MOESM1_ESM.pdf]

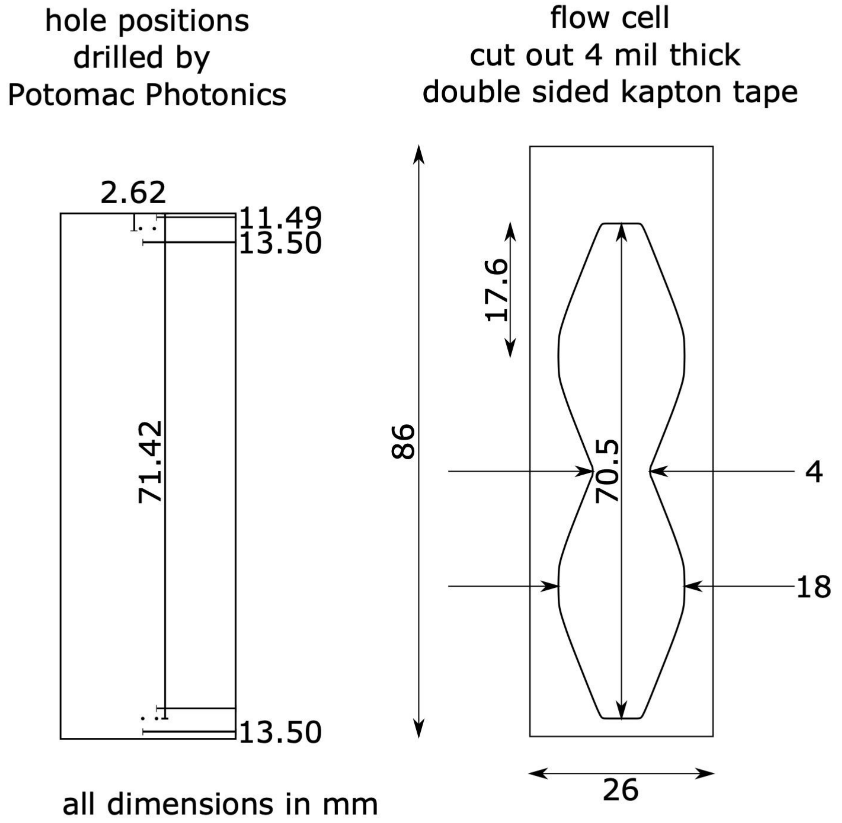

Supplementary Figure 1 Design of a PySeq2500 flow cell compatible with the HiSeq2500 stage and integrated fluidics (A) Positions of 0.5 mm diameter through holes on a 25 x 75 mm glass slide which align with the 2 hole stage inlets and outlets 4 & 5 on the HiSeq 2500 stage. (B) PySeq2500 flow cell chamber dimensions. Flow cell chambers are manufactured using a Cricut cutting machine from 4 mil thick polyimide tape with double sided adhesive. Excess polyimide tape is trimmed to the edge of the slide before flow cell assembly. (all dimensions in mm)

Supplementary Appendix 1

```
# Experiment Details
[experiment]
method = 4i         # name of installed method (string), section with HiSeq settings (string),
                    # or path to method, config file (path)
cycles = 6          # number of cycles to run (integer)
first flowcell = A  # which flowcell to run first, A or B (string), default is 'A'

# Position of Sections on Flowcell
# section name = AorB: LLx, LLy, URx, URy (use slide grid)
#       - section name: Name of area on slide to image
#       - LLx: Lower left x position on flowcell
#       - LLy: Lower left x position on flowcell
#       - URx: Upper right x position on flowcell
#       - URy: Upper right y position on flowcell
[sections]
CU59_SCL_A1 = A: 17, 23.5, 8, 13.5
NEUKGB38WER_SCL_A2 = A: 18, 60, 8.5, 50
CU59_B1 = B: 17, 25, 8.5, 14.5
NEUKGB38WER_SCL_B2 = B: 18, 58.5, 8.5, 49.5

# Specify Cycle Specific Reagents
# variable_reagent N = reagent name
#       -variable_reagent: cycle dependent reagent in recipe, must be lowercase and no spaces (string)
#       -N: cycle (integer)
#       -reagent name: reagent used for variable_regent at cycle N (string)
[cycles]
1stab 1 = LMN1B+CD34
1stab 2 = CD68+TDP43+MAP2
1stab 3 = ELAVL3+pTDP43+NFH
1stab 4 = TMEM119+GFAP+IBA1
1stab 5 = MBP+ALDH1L1
1stab 6 = P62+AQP4
2ndab 1 = MS594+SHPCY5
2ndab 2 = MS532+RB594+CHKCY5
2ndab 3 = MS532+RB594+CHKCY5
2ndab 4 = MS594+CHKCY5+RB700
2ndab 5 = MS594+RB647
2ndab 6 = MS594+RB647

# Specify Filters to Use for Each Cycle
#
# color N = name
#       -color: laser color (string, ie green, g, or G)
#       -N: cycle (integer)
#       -name: filter used for imaging at cycle N, see table below for options (float/string)
#
#       ===========  ===========  ===========================================
#       laser color  laser index          filters (Optical Density)
#       ===========  ===========  ===========================================
#       green             1          open, 1.0, 2.0, 3.5, 3.8, 4.0, 4.5, home
#       red               2          open, 0.2, 0.5, 0.6, 1.0, 2.4, 4.0, home
#       ===========  ===========  ===========================================
#
```

```
#  For Example:
#  green 1: 1.6
#
[filters]

# Assign Reagents to Ports
# N = name
#       -N: port number (integer)
#       -name: name of reagent
[reagents]
1 = PBS
3 = water
2 = elution
12 = blocking
13 = imaging
9 = LMN1B+CD34
20 = CD68+TDP43+MAP2
21 = ELAVL3+pTDP43+NFH
22 = TMEM119+GFAP+IBA1
23 = MBP+ALDH1L1
24 = P62+AQP4
14 = MS594+SHPCY5
15 = MS532+RB594+CHKCY5
16 = MS594+CHKCY5+RB700
17 = MS594+RB647


# Method specific section (optional)
#
# Can use installed methods or seperate method config file instead
# To see installed methods, pyseq -l
# To see details of installed methods, pyseq -m method_name
# To see configuration options, pyseq -s
[4i]
recipe = 4i_recipe.txt                # path to the method recipe (path), required
reagent flowrate = 100                # flow rate to pump reagents during recipe in uL/min (integer), default is the
                                        `minimum flow rate`
variable reagents = 1stab, 2ndab      # name of variable ports in recipes that are cycle dependent (string)
first port = blocking                 # port to start recipe at on first cycle (string)
rinse = PBS                           # neutral reagent to rinse the experimental flow cell (string), default is `None`
barrels per lane = 8                  # number of syringe barrels that are used per lane on flow cell (integer),
                                        default is 1
red laser power = 225                 # set power of red laser in mW (integer), default is 10
green laser power = 225               # set power of green laser in mW (integer), default is 10
red focus filter = home               # filter for red laser for autofocus routine, default is 2.0
green focus filter = 1.0              # filter for green laser for autofocus routine, default is 2.0
default green filter = open           # filter for green laser if not specified in **[filter]** section of experiment
                                        config file (float/string), default is `home`
default red filter = open             # filter for red laser if not specified in **[filter]** section of experiment config
                                        file (float/string), default is `home`
default em filter = False             # default em filter = emission filter used for imaging, True for in path, False
                                        for out of path (bool), default is True
autofocus = partial once              # routine used for autofocusing (string), see **Autofocus** for more info,
                                        default is `partial once`
```

focus tolerance = 10	# distance in microns for acceptable focus error, default is 0 um which allows for maximum error
z planes = 10	# z planes = override number of z planes to image (integer) in recipe
z position = 21500	# step of tilt motors when imaging (integer), default is 21500
overlap = 256	# column pixel overlap (int) for adjacent tiles in x direction, default is 0
overlap direction = left	# overlap direction = side of image to overlap (str), either left or right, default is left
flush flowrate = 5000	# flowrate to flush lines with in uL/min (integer), default is 700
prime flowrate = 1000	# flowrate to prime lines with in uL/min (integer), default is 100
pre recipe = pre_imaging.txt	# path to the recipe to perform before the main experiment (path)

Supplementary Appendix 2

recipe = path to the method recipe as its value (path), required
flush volume = volume to flush line with in uL (integer), default is 1000
main prime volume = volume to prime main lines (ports 1-8 & 10-19) in uL (integer), default is 500
side prime volume = volume to prime side lines (ports 9 & 22-24) in uL (integer), default is 350
sample prime volume = volume to prime samples lines (port 20) in uL (integer), default is 250
flush flowrate = flowrate to flush lines with in uL/min (integer), default is 700
prime flowrate = flowrate to prime lines with in uL/min (integer), default is 100
reagent flowrate = flowrate to pump reagents during recipe in uL/min (integer), default is the `minimum flow rate`
variable reagents = name of variable ports in recipes that are cycle dependent (string)
first port = port to start recipe at on first cycle (string)
barrels per lane = number of syringe barrels that are used per lane on flowcell (integer), default is 1
inlet ports = 2 inlet port row or 8 inlet port row (integer), default is 2
side ports = ports not plumbed for use in the chiller, default are 9, 21, 22, 23, 24
sample port = port plumbed to sample tubes in stage area, default is 20
green laser power = set power of green laser in mW (integer), default is 10
red laser power = set power of red laser in mW (integer), default is 10
green focus filter = filter for green laser for autofocus routine, default is 2.0
red focus filter = filter for red laser for autofocus routine, default is 2.0
default em filter = emission filter used for imaging, True for in path, False for out of path (bool), default is True
default green filter = filter for green laser if not specified in **[filter]** section of experiment config file (float/string), default is `home`
default red filter = filter for red laser if not specified in **[filter]** section of experiment config file (float/string), default is `home`
rinse = reagent to rinse the flowcell with between completion of the experiment and flushing of the lines during shutdown (string), default is `None`
overlap = column pixel overlap (int) for adjacent tiles in x direction, default is 0
overlap direction = side of image to overlap (str), either left or right, default is left
z position = step of tilt motors when imaging (integer), default is 21500
autofocus = routine used for autofocusing (string), see **Autofocus** for more info, default is `partial once`
focus tolerance = distance in microns for acceptable focus error, default is 0 um which allows for maximum error
focus range = percent of objective range (float) to use for autofocus, default is 100
focus spacing = distance in microns between frames in an objective stack, default is 0.5
enable z stage = enable/disable z stage movements (bool), default is True.
temperature interval = interval time in minutes (float) to query flowcell temperature, default is 5
bundle height = sensor bundle height of cameras (integer), only certain values are valid, default is 128
speed up = factor to decrease hold times (integer) for a virtual HiSeq experiment, default is 5000.
z planes = override number of z planes to image (integer) in recipe
stack split = portion of stack below optimal focus plane (float), default is 2/3
pre recipe = path to the recipe to perform before the main experiment (path)

Supplementary Appendix 3

```
PORT:    water        #Move valve to water(port 2)
PUMP:    2000         #Pump 500 uL
PORT:    elution      #Move valve to elution (port 3)
PUMP:    500          #Pump 500 uL
HOLD:    10           #Hold for 10 minutes
PORT:    elution      #Move valve to elution (port 3)
PUMP:    500          #Pump 500 uL
HOLD:    10           #Hold for 10 minutes
PORT:    elution      #Move valve to elution (port 3)
PUMP:    500          #Pump 500 uL
HOLD:    10           #Hold for 10 minutes
PORT:    elution      #Move valve to elution (port 3)
PUMP:    500          #Pump 500 uL
HOLD:    10           #Hold for 10 minutes
PORT:    elution      #Move valve to elution (port 3)
PUMP:    500          #Pump 500 uL
HOLD:    10           #Hold for 10 minutes
PORT:    water        #Move valve to water(port 2)
PUMP:    2000         #Pump 500 uL
PORT:    blocking     #Move valve to blocking buffer (port 4)
PUMP:    800          #Pump 800 uL
HOLD:    60           #Hold for 60 min
PORT:    elution      #Move to elution for timing purpose
PORT:    PBS          #Move valve to PBS wash (port 1)
PUMP:    2000         #Pump 2000 uL
PORT:    1stab        #Move valve to primary antibody (variable)
PUMP:    500          #Pump 500 uL
HOLD:    120          #Hold for 120 min
PORT:    elution      #Move to elution for timing purpose
PORT:    PBS          #Move valve to PBS wash (port 1)
PUMP:    2000         #Pump 2000 uL
PORT:    2ndab        #Move valve to secondary antibody (variable)
PUMP:    1000         #Pump 500 uL
HOLD:    120          #Hold for 120 min
PORT:    elution      #Move to elution for timing purpose
PORT:    PBS          #Move valve to PBS wash (port 1)
PUMP:    2000         #Pump 2000 uL
WAIT:    elution      #Wait till other flowcell is eluting with elution buffer
PORT:    imaging      #Move valve to imaging buffer (port 5)
PUMP:    750          #Pump 750 uL
IMAG:    15           #image 15 z sections
```

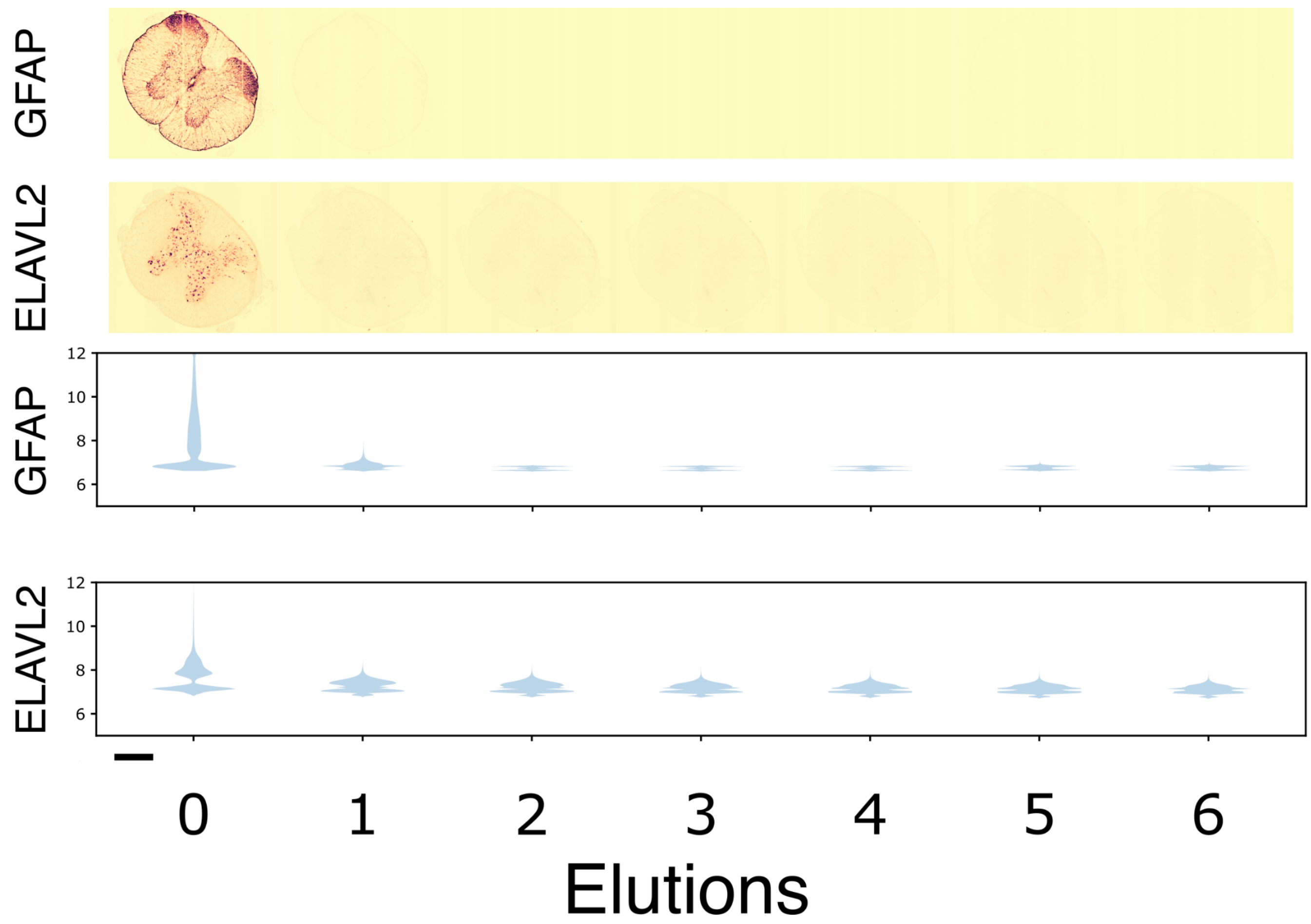

Supplementary Figure 2 Automated elution series for optimizing 4i protocol for use on fresh frozen tissue sections. (*top*) A mouse spinal cord section was stained for GFAP (687 nm channel) and ELAVL2 (610 nm channel) and imaged after sequential rounds of elution. (*bottom*) Histograms of 10^6 random pixels from each round of elution. Y axis represents log2(pixel intensity).

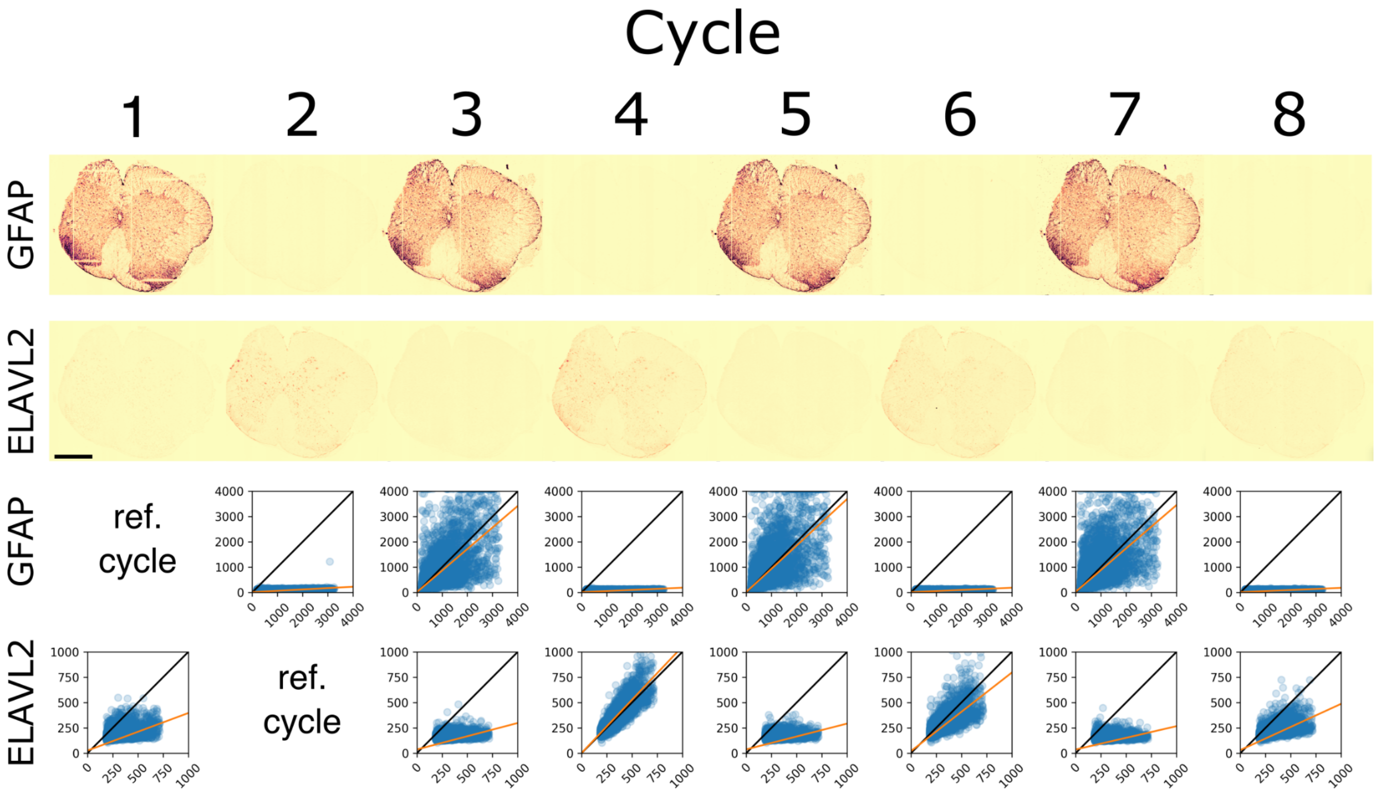

Supplementary Figure 3 Mouse spinal cord section was immunostained with Chicken anti-GFAP in odd cycles, Rabbit anti-ELAVL2 in even cycles using our PySeq2500 automated 4i protocol. The same secondary antibody cocktail, Cy5 donkey anti-Chicken and AF594 Donkey anti-Rabbit was used in each of the 8 cycles. (*top*) Images of GFAP (687 nm channel) and ELAVL2 (610 nm channel) from each cycle of staining (scale = 500 µm). (*bottom*) Randomly subsampled (10^5 pixels) pixel intensities from the reference cycle vs all other cycles. The first cycle of staining with each primary antibody, cycles 1 and 2 for GFAP (687 nm channel) and ELAVL2 (610 nm channel) respectively, was used as the reference cycle. All pixels fit to a line weighted by pixel intensity for each cycle is shown as an orange line and y = x is shown as a black line.

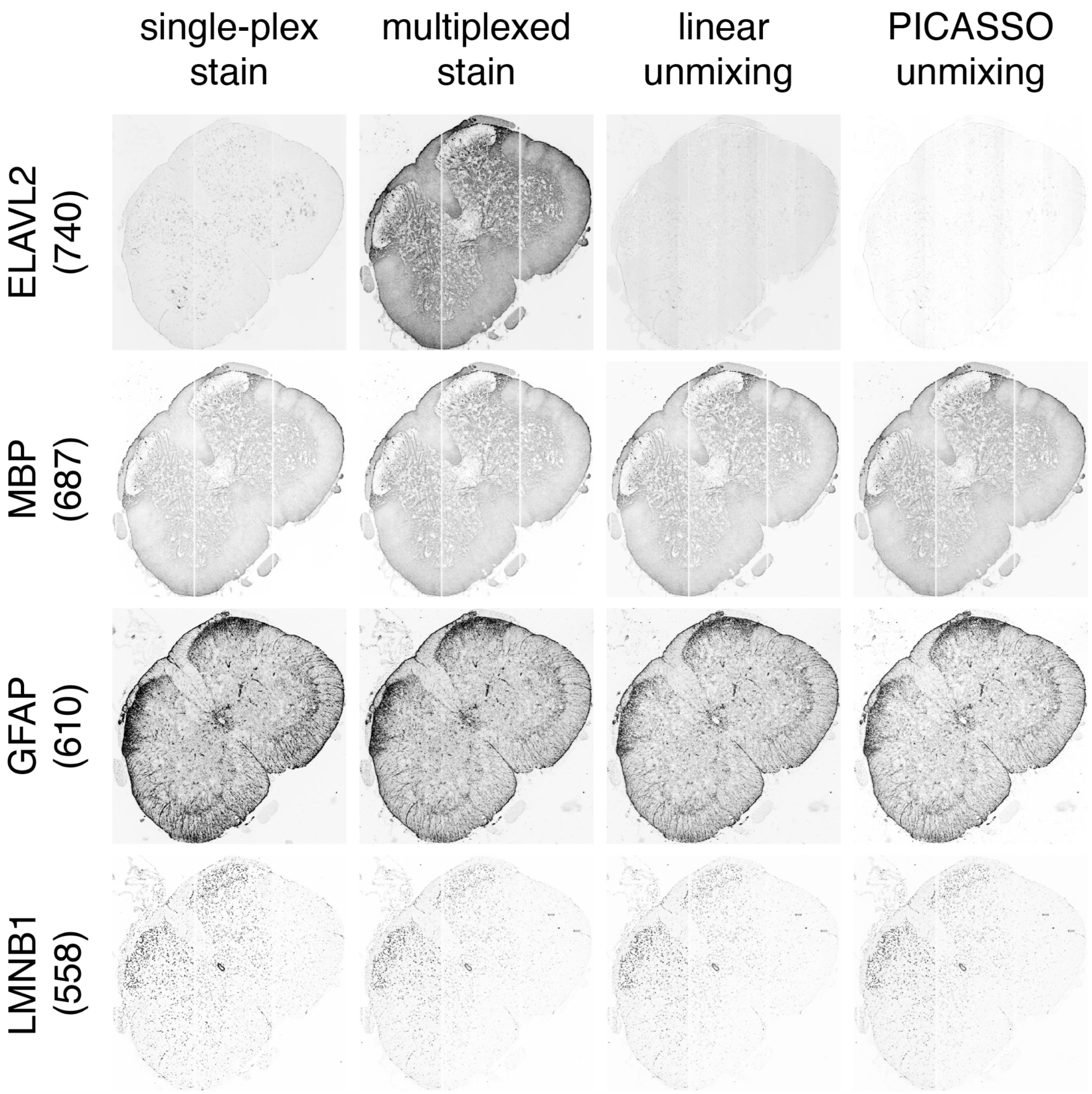

Supplementary Figure 4 Mouse spinal cord section stained in singleplex cycles (cycles 1-4) and in the mixed multiplexed cycle (cycle 5). Individual stains from the multiplexed cycle (cycle 5) calculated using linear unmixing and PICASSO unmixing strategies.

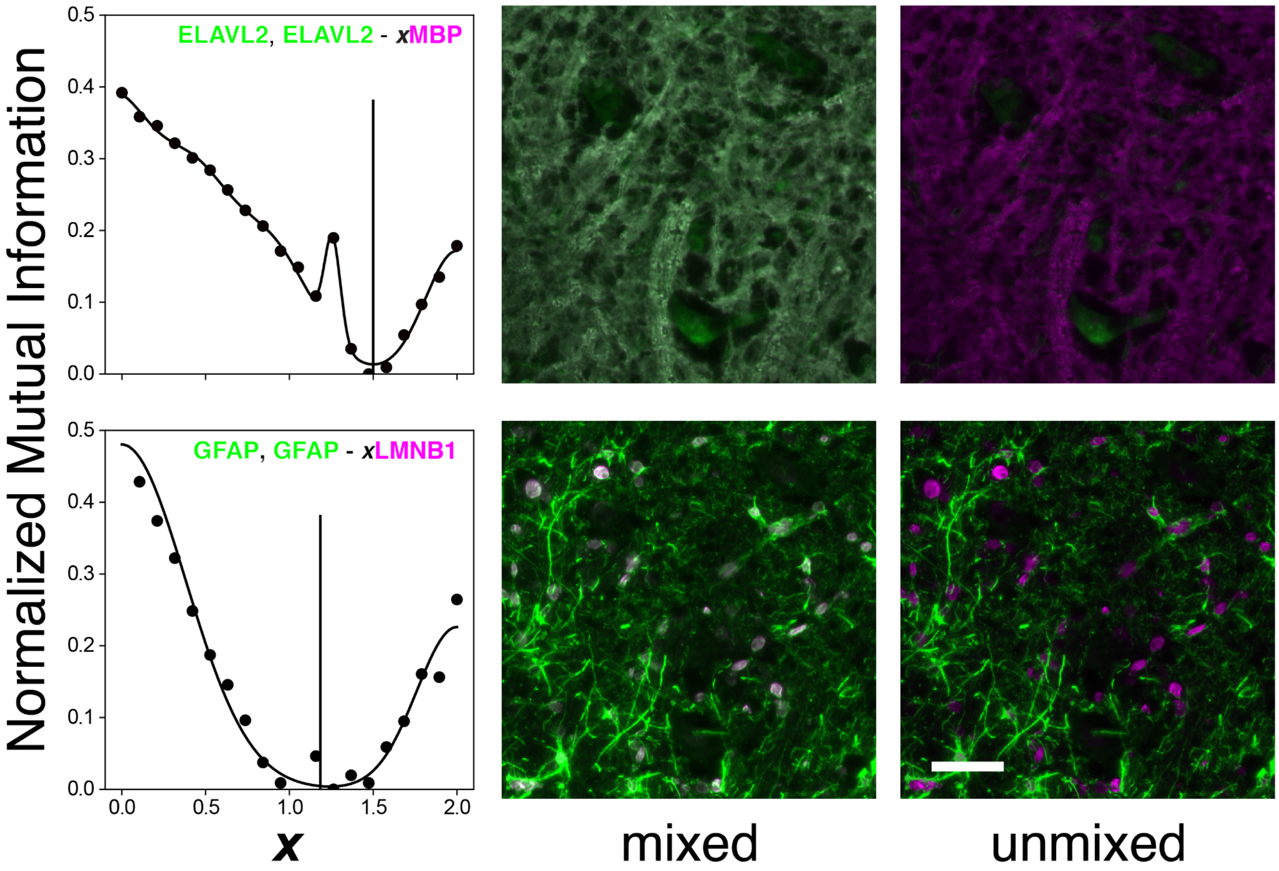

Supplementary Figure 5 PICASSO unmixing by minimization of mutual information. Mutual information, ***I***, was calculated at a range of relative leakage, ***x***, from (*top*) MBP into ELAVL2 and (*bottom*) LMNB1 into GFAP. The optimal ***x*** was estimated as the minimal point of a curve fit to ***I***(***x***) and used to unmix the spillover signal. Scale bar represents 100 um.

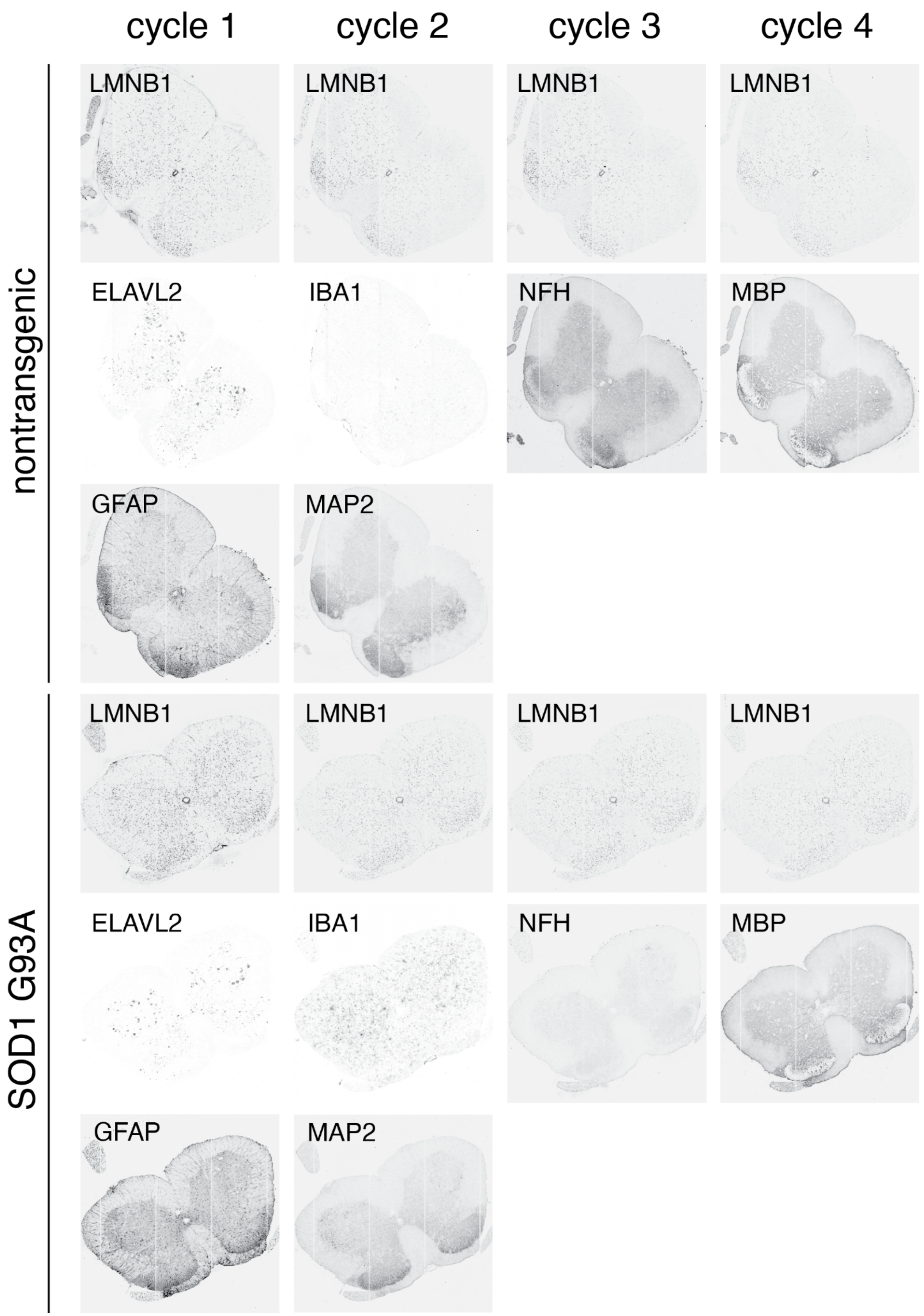

Supplementary Figure 6 Fresh frozen spinal cord sections from nontransgenic and SOD1 G93A mutant mice were subjected to four cycles of 4i to visualize six cell type markers, with LMNB1 in each cycle.

FIGURE	TISSUE	4i CYCLE	PRIMARY	VENDOR	CATALOG #	CLONE	HOST	DILUTION	LOT #	SECONDARY	VENDOR	CATALOG #	DILUTION	LOT#
Supplemental figure 2	WT p70, lumbar L1-L3, fresh frozen	Cycle 1	GFAP	Abcam	ab4674	polyclonal	Chicken	1:500	GR3368950-1	Cy5 Donkey Anti-Chicken	Jackson	703-175-155	1:500	130611
			ELAVL2	Atlas	HPA063001	polyclonal	Rabbit	1:100	000015755	Alexa Fluor 594 Donkey Anti-Rabbit	Jackson	711-585-152	1:500	140419
Supplemental figure 3	WT p70, lumbar L1-L3, fresh frozen	Odd cycles	GFAP	Novus	NBP1-05198	polyclonal	Chicken	1:500	7529-18	Cy5 Donkey Anti-Chicken	Jackson	703-175-155	1:500	130611
										Alexa Fluor 594 Donkey Anti-Rabbit	Jackson	711-585-152	1:500	140419
		Even cycles	ELAVL2	Atlas	HPA063001	polyclonal	Rabbit	1:100	000015755	Cy5 Donkey Anti-Chicken	Jackson	703-175-155	1:500	130611
										Alexa Fluor 594 Donkey Anti-Rabbit	Jackson	711-585-152	1:500	140419
Figure 2, Supplemental figures 4-5	WT p30, lumbar L1-L3, fresh frozen	Cycle 1	GFAP	Abcam	ab4674	polyclonal	Chicken	1:500	GR3368950-2	Alexa Fluor 594 Goat Anti-Chicken	Invitrogen	A11042	1:500	673785
		Cycle 2	ELAVL2	Atlas	HPA063001	polyclonal	Rabbit	1:100	000015755	Alexa Fluor 700 Goat Anti-Rabbit	Invitrogen	A21038	1:500	2129003
		Cycle 3	LMNB1	Sigma	AMAB91251	CL3929	Mouse	1:100	MAB-03076	Alexa Fluor 532 Goat Anti-Mouse	Invitrogen	A11002	1:500	2160043
		Cycle 4	MBP	Abcam	ab209328	IGX3421	Human	1:1000	GR3215004-10	Cy5 Donkey Anti-Human	Jackson	709-175-149	1:500	129880
		Cycle 5	LMNB1	Sigma	AMAB91251	CL3929	Mouse	1:100	MAB-03076	Alexa Fluor 532 Goat Anti-Mouse	Invitrogen	A11002	1:500	2160043
			GFAP	Abcam	ab4674	polyclonal	Chicken	1:500	GR3368950-2	Alexa Fluor 594 Goat Anti-Chicken	Invitrogen	A11042	1:500	673785
			MBP	Abcam	ab209328	IGX3421	Human	1:1000	GR3215004-10	Cy5 Donkey Anti-Human	Jackson	709-175-149	1:500	129880
			ELAVL2	Atlas	HPA063001	polyclonal	Rabbit	1:100	000015755	Alexa Fluor 700 Goat Anti-Rabbit	Invitrogen	A21038	1:500	2129003
Figure 3, Supplemental figure 6	M387, NTG p100 male; M388 SOD1 G93A p100 male	Cycle 1	LMNB1	Sigma	AMAB91251	CL3929	Mouse	1:200	MAB-03076	Alexa Fluor 532 Goat Anti-Mouse	Invitrogen	A11002	1:500	2160043
			ELAVL2	Atlas	HPA063001	polyclonal	Rabbit	1:100	000015755	Alexa Fluor 594 Donkey Anti-Rabbit	Jackson	711-585-152	1:1000	151658
			GFAP	Abcam	ab4674	polyclonal	Chicken	1:500	GR3368950-3	Cy5 Donkey Anti-Chicken	Jackson	703-175-155	1:1000	152944
		Cycle 2	LMNB1	Sigma	AMAB91251	CL3929	Mouse	1:200	MAB-03076	Alexa Fluor 532 Goat Anti-Mouse	Invitrogen	A11002	1:500	2160043
			IBA1	Abcam	ab178847	EPR16589	Rabbit	1:250	GR3229566-15	Alexa Fluor 594 Donkey Anti-Rabbit	Jackson	711-585-152	1:1000	151658
			MAP2	Abcam	ab5392	polyclonal	Chicken	1:500	GR3386708-1	Cy5 Donkey Anti-Chicken	Jackson	703-175-155	1:1000	152944
		Cycle 3	LMNB1	Sigma	AMAB91251	CL3929	Mouse	1:200	MAB-03076	Alexa Fluor 532 Goat Anti-Mouse	Invitrogen	A11002	1:500	2160043
			PVALB	Swant	PV27a	polyclonal	Rabbit	1:1000	NA	Alexa Fluor 594 Donkey Anti-Rabbit	Jackson	711-585-152	1:1000	151658
			NfH	Abcam	ab4680	polyclonal	Chicken	1:250	GR3359372-1	Cy5 Donkey Anti-Chicken	Jackson	703-175-155	1:1000	152944
		Cycle 4	LMNB1	Sigma	AMAB91251	CL3929	Mouse	1:200	MAB-03076	Alexa Fluor 532 Goat Anti-Mouse	Invitrogen	A11002	1:500	2160043
			PDGFRa	Cell Signaling	3174S	D1E1E	Rabbit	1:500	8	Alexa Fluor 594 Donkey Anti-Rabbit	Jackson	711-585-152	1:1000	151658
			MBP	Abcam	ab209328	IGX3421	Human	1:1000	GR3215004-10	Cy5 Donkey Anti-Human	Jackson	709-175-149	1:500	129880
Figure 4	sporadic ALS patients	Cycle 1	CD34	R&D Systems	AF7227		Sheep	1:50	CFZV0121051	Cy5 Donkey Anti-Sheep	Jackson	713-175-147	1:1000	152945
			LMNB1	Sigma	AMAB91251	CL3929	Mouse	1:100	MAB-03502	Alexa Fluor 594 Donkey Anti-Mouse	Jackson	715-585-150	1:1000	153991
		Cycle 2	CD68	DAKO	M0814	KP1	Mouse	1:100	41258320	Alexa Fluor 532 Goat Anti-Mouse	Invitrogen	A11002	1:500	2160043
			TDP-43	Proteintech	10782-2-AP	polyclonal	Rabbit	1:250	00065465	Alexa Fluor 594 Donkey Anti-Rabbit	Jackson	711-585-152	1:1000	151658
			MAP2	Abcam	ab5392	polyclonal	Chicken	1:500	GR3386708-1	Cy5 Donkey Anti-Chicken	Jackson	703-175-155	1:1000	152944
		Cycle 3	ELAVL3	Invitrogen	A-21271	16A11	Mouse	1:250	2105721	Alexa Fluor 532 Goat Anti-Mouse	Invitrogen	A11002	1:500	2160043
			pTDP-43	Proteintech	22309-1-AP	polyclonal	Rabbit	1:250	00058641	Alexa Fluor 594 Donkey Anti-Rabbit	Jackson	711-585-152	1:1000	151658
			NFH	Abcam	ab4680	polyclonal	Chicken	1:250	GR3359372-4	Cy5 Donkey Anti-Chicken	Jackson	703-175-155	1:1000	152944
		Cycle 4	TMEM119	Atlas	AMAb91528	CL8714	Mouse	1:250	MAB-03453	Alexa Fluor 594 Donkey Anti-Mouse	Jackson	715-585-150	1:1000	153991
			IBA1	Abcam	ab178847	EPR16589	Rabbit	1:250	GR3229566-20	Alexa Fluor 700 Goat Anti-Rabbit	Invitrogen	A21038	1:500	2129003
			GFAP	Abcam	ab4674	polyclonal	Chicken	1:500	GR3393187-1	Cy5 Donkey Anti-Chicken	Jackson	703-175-155	1:1000	152944
		Cycle 5	MBP	Atlas	AMAb91062	CL2819	Mouse	1:500	MAB02927	Alexa Fluor 594 Donkey Anti-Mouse	Jackson	715-585-150	1:1000	153991
			ALDH1L1	Atlas	HPA050139	polyclonal	Rabbit	1:1000	000016054	Alexa Fluor 647 Donkey Anti-Rabbit	Jackson	711-605-152	1:1000	154880
		Cycle 6	P62	Abcam	ab56416	polyclonal	Mouse	1:100	GR3294261-1	Alexa Fluor 594 Donkey Anti-Mouse	Jackson	715-585-150	1:1000	153991
			AQP4	Atlas	HPA014784	polyclonal	Rabbit	1:1000	000014721	Alexa Fluor 647 Donkey Anti-Rabbit	Jackson	711-605-152	1:1000	154880

Table 1 Primary and secondary antibodies used in automated 4i experiments.
